# 

**Published:** 1921-06-04

**Authors:** 


June 4, 1921] [Price Twopence

### The Hospital

The Workers' Newspaper of Administrative Medicine and Institutional Life, Administration, National Insurance and Health.

### CONTENTS

Closing of Wards at the London Hospital ... 159
Harley Street ... ... ... ... ... ... 159
Mr. Keyser's Proposal ... ... ... ... ... 160
The Federation of Medical and Allied Societies 160
Notes of the Week ... ... ... ... ... 161
Lord Cave's Commission—The International Tuberculosis Conference—£4,000 for X-ray Research—£10,000 a year for the Middlesex—Liability to Industrial Disease —The Prince and the Miners' Hospital—Gift of a Page in "Punch"—Insured Patients and Hospitals—A Pageant in Edinburgh—Paying Patients at Camberwell Infirmary—Hospital Secretary Joins the Board—Cost of Maintenance and Gifts in Kind.
Lunacy and Litigiousness ... ... ... ... 163
The Moral of Recent Experience.
Dangerous Drugs Act New Regulations ... ... 163
The Departmental Committee's Report.
Asthma and Hay Fever ... ... ... ... 165
The New Methods of Treatment.
The Search for the Soul ... ... ... ... 166
The Public Well-Being ... ... ... ... ... 167
Fighting a Universal Plague—Educational Films.
The Matrons' and Sisters' Department ... ... 169
The Registered Children's Nurse.
Two Methods of Nursing Insured Persons.
Round the Hospitals ... ... ... ... ... 170
The Editor's Letter-Box ... ... ... ... 172
The Hospital Pageant Meeting—An Appreciation.
Passing Events and Topics ... ... ... ... 172
The National Relief Fund ... ... ... ... 172
The Position at the London Hospital ... ... vi
Representation for Poor-Law Pharmacists ... vi
The College of Nursing (Limited by Guarantee) viii
Matrons' and Sisters' Appointments ... ... viii

